# Total bilirubin as a marker for hemolysis and outcome in patients with severe ARDS treated with veno-venous ECMO

**DOI:** 10.1186/s12871-025-02988-1

**Published:** 2025-03-13

**Authors:** Victoria Bünger, Mario Menk, Oliver Hunsicker, Alexander Krannich, Felix Balzer, Claudia D. Spies, Wolfgang M. Kuebler, Steffen Weber-Carstens, Jan A. Graw

**Affiliations:** 1 Department of Anesthesiology and Intensive Care Medicine, CCM / CVK Charité, Universitätsmedizin Berlin, corporate member of Freie Universität Berlin, Humboldt-Universität Zu Berlin, Augustenburger Platz 1, Berlin, 13353 Germany; 2 ARDS/ECMO Centrum Charité, Charité–Universitätsmedizin Berlin, Berlin, Germany; 3 Department of Anesthesiology and Intensive Care Medicine, University Hospital “Carl Gustav Carus”, Technische Universität Dresden, Dresden, Germany; 4 Experimental and Clinical Research Center (ECRC), Charité–Universitätsmedizin Berlin, Berlin, Germany; 5 BioStats GmbH, Nauen, Germany; 6 Institute of Medical Informatics, Charité– Universitätsmedizin Berlin, Berlin, Germany; 7 Institute of Physiology, Charité – Universitätsmedizin Berlin, Berlin, Germany; 8 Department of Anesthesiology and Intensive Care Medicine, Universitätsklinikum Ulm, Ulm University, Ulm, Germany

**Keywords:** Total bilirubin, Hemolysis, Extracorporeal membrane oxygenation, Acute lung injury, Hyperbilirubinemia

## Abstract

**Background:**

Hemolysis is a common complication in critically ill patients with sepsis, acute respiratory distress syndrome (ARDS) or therapy with extracorporeal membrane oxygenation (ECMO). Heme degradation product bilirubin might accumulate in conditions of significant hemolysis. In patients with ARDS and therapy with veno-venous ECMO (vvECMO), the prognostic potential of elevated initial total bilirubin (tBili) was investigated.

**Methods:**

Retrospective analysis of patients with ARDS and vvECMO-therapy (*n* = 327) admitted to a tertiary ARDS center. A tBili cut-off value was determined by binary recursive partitioning. Baseline characteristics were compared and relevant variables were included in a multivariate logistic regression model with backward variable selection. Primary endpoint was survival within 28 days analyzed with Kaplan–Meier-curves and cox regression. Secondary endpoints included failure free composites for organ dysfunction, renal replacement therapy (RRT), vasopressor therapy and ECMO within 28 days and were compared using competing risk regression analysis.

**Results:**

A cut-off value of 3.6mg/dl divided the cohort for ICU mortality (tBili ≤ 3.6mg/dl: 46% (*n* = 273) vs. tBili > 3.6mg/dl: 78% (*n* = 54), *p* < 0.001). The group with tBili > 3.6mg/dl showed a higher 28-day mortality (HR 3.03 [95%CI 2.07–4.43], *p* < 0.001) and significantly lower chances of successful recovery from organ dysfunction (subdistribution hazard ratio (SHR) 0.29 [0.13–0.66], *p* < 0.001), RRT (SHR 0.34 [0.14–0.85], *p* = 0.02), and ECMO (SHR 0.46 [0.25–0.86], *p* = 0.015) compared to the group with tBili ≤ 3.6mg/dl. Recovery from vasopressor therapy did not differ between groups (SHR 0.63 [0.32–1.24], *p* = 0.18).

**Conclusion:**

Patients with ARDS, vvECMO-therapy and tBili > 3.6mg/dl had a higher mortality and lower chances for recovery from organ dysfunction, RRT, and ECMO within 28 days. The tBili-cut-off value may be useful to identify patients at risk for unfavorable outcomes.

**Supplementary Information:**

The online version contains supplementary material available at 10.1186/s12871-025-02988-1.

## Background

Hemolysis is a common complication in critically ill patients with sepsis, the acute respiratory distress syndrome (ARDS), or therapy with extracorporeal membrane oxygenation (ECMO) [1–5]. Upon hemolysis, the hepatic glycoprotein haptoglobin (Hp) and cell-free hemoglobin (CFH) form a Hp-CFH-complex which binds to the CD163-receptor of macrophages and is removed from plasma via endocytosis by the spleen or the liver [6–8]. Heme oxygenase 1 (HO-1) degrades the heme functional group into free iron, carbon monoxide, and biliverdin [9]. The liver processes biliverdin further to unconjugated bilirubin and finally water-soluble conjugated bilirubin. As an end-product of hemoglobin catabolism, elevated plasma concentrations of bilirubin are therefore not only detected in liver dysfunction but also in conditions of increased hemolysis [10, 11].

ARDS is a life-threatening clinical syndrome affecting about 10% of intensive care unit (ICU) patients and associated with considerable mortality [12]. In critically ill patients with ARDS and sepsis, bilirubin plasma concentrations on ICU admission correlate with 60-day mortality [13]. Similarly, bilirubin plasma concentrations in the initial phase of ARDS are associated with mortality [14, 15]. In addition, several small retrospective studies in patients with ARDS and treatment with ECMO reported an association between elevated bilirubin plasma levels and mortality [16–18]. While some studies focused on the initial bilirubin plasma concentration patients with ARDS on ICU admission, the study of Lazzeri and colleagues also included data on bilirubin plasma concentration kinetics during the first three days of treatment with ECMO [16, 18]. Further studies investigated the relevance of peak plasma concentrations of bilirubin in connection with 28-day mortality in patients with ARDS and treatment with ECMO [17]. Ample data exist on adverse outcomes associated with compromised liver function in patients treated with veno-arterial EMCO for compromised cardiopulmonary function but only few studies have reported comparable data in patients treated exclusively with veno-venous ECMO for support of pulmonary function [16, 19, 20].

In patients with ARDS and treatment with veno-venous ECMO, elevated plasma concentrations of CFH are known to be associated with increased ICU-mortality and organ failure [4, 5]. Serum bilirubin may not only act as a marker of disease severity and hemolysis, but has also been proposed to contribute to disease progression in ARDS patients, e.g. by direct toxic effects of bilirubin on lung surfactant [21–23]. As a downstream product of CFH metabolism, bilirubin plasma concentration might be affected not only by the underlying disease but also by the degree of hemolysis induced by extracorporeal circulation. In this study, the prognostic potential of elevated initial total bilirubin (tBili) concentrations for survival and recovery from organ dysfunctions including successful weaning from organ replacement therapies in patients with ARDS and therapy with veno-venous ECMO was investigated.

## Methods

### Study design and setting

This study was performed in a previously described cohort of patients with ARDS admitted to a tertiary ARDS referral center which serves as a national reference center for therapy of severe ARDS [24]. The study included patients with ARDS receiving treatment with veno-venous ECMO and measurements of CFH and Hp values within the first seven days of ECMO treatment [24]. The study was approved by the Medical Ethics Committee of the Charité*–*Universitätsmedizin Berlin (No. EA2/019/19).

### Participants and grouping

**Fig. 1:**
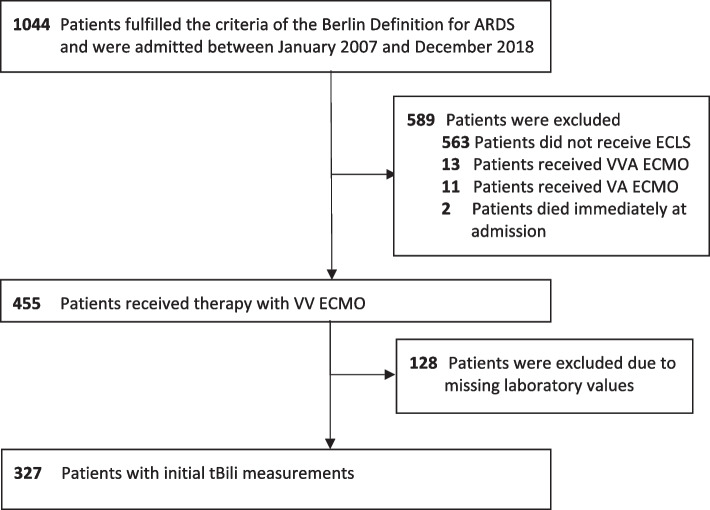
Consort diagram. ECLS – extracorporeal life support; ECMO – extracorporeal membrane oxygenation with veno-veno-arterial (V-VA), venoarterial (V-A), or venovenous (V-V) cannulation

All patients fulfilling the criteria for ARDS according to the Berlin Definition and admitted to our ARDS center between January 2007 and December 2018 were included in the study (Fig. 1). Patients not receiving treatment with veno-venous ECMO for pulmonary organ replacement such as patients receiving no extracorporeal life support (ECLS) or patients receiving ECMO with veno-arterial or veno-veno-arterial configuration were excluded from analyses. Furthermore, patients moribund on admission and patients without measured plasma concentrations of tBili at ECMO initiation (initial tBili) were excluded (Fig. 1). Eligible patients were grouped according to a calculated cut-off value for initial tBili as specified in the statistical analysis section [24].

### Data sources

Patient data and laboratory values were extracted from the two electronic patient data management systems used at the hospital (SAP, Walldorf, Germany and COPRA, Sasbachwalden, Germany) [25]. CFH and Hp plasma concentrations were measured as described previously [4, 5].

### Endpoints

The primary endpoint was ICU mortality within 28 days after ECMO initiation. Failure-free composites were used as secondary endpoints as previously reported based on the re-appraisal of composite outcomes in critical care patients [25, 26]. In brief, secondary endpoints comprised the following endpoints: “free from organ dysfunction”, “free from renal replacement therapy (RRT)”, “free from vasopressors”, and “free from ECMO” within 28 days after ECMO initiation [25, 27].

### Statistical analysis

The cut-off value for initial tBili regarding a significant increase in ICU mortality was calculated by binary recursive partitioning. Baseline characteristics between the groups were compared. Differences of continuous variables were tested for significance by Mann–Whitney-U test, differences in frequencies by Fisher´s test as appropriate. A two-tailed *p* value < 0.05 was considered significant. All significantly different variables were included in a multivariate logistic regression model for ICU mortality with backward variable selection according to AIC. Only complete cases were included in the analysis. For the primary endpoint of 28-day survival, Kaplan–Meier-curves and cox regression were analyzed and tested for significance by log-rank test. Analogously, secondary endpoints were compared with competing risk regression analysis and significance was tested by log-rank test. For comparison of tBili values over time, ANOVA with post-hoc test and daily comparison of tBili values between the groups with Mann–Whitney-U test and Bonferroni correction was performed. Statistical analysis was performed with R Version 4.2.2.

## Results

**Fig. 2:**
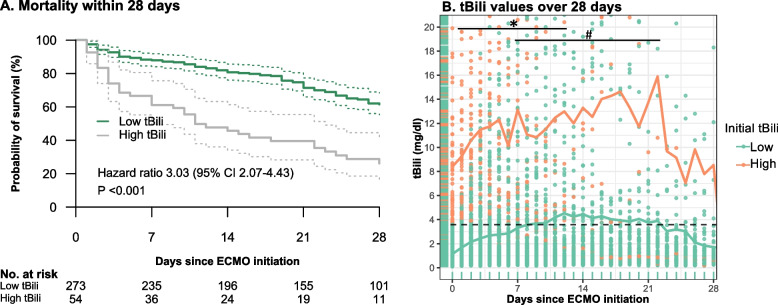
**A** Kaplan Meyer Survival Curves for mortality within 28 days (Hazard Ratio 3.03 (95% CI 2.07–4.43), *P* < 0.001). **B** Total Bilirubin (tBili) concentration over 28 days after ECMO initiation. Points represent individual tBili values. Lines represent mean values. Green – low initial tBili, orange – high initial tBili. * indicates significantly different values between both groups (Mann–Whitney-U test with Bonferroni correction, *p* < 0.05). # indicates significantly different tBili values from initial tBili in the low initial tBili group (ANOVA and post-hoc test, *p* < 0.05). In the high initial tBili group the differences in tBili over time from initial value were not significant

In total, 327 patients with initial tBili values were included in the analysis (Supplemental Fig. 1). A cut-off value for initial tBili of 3.6 mg/dl was associated with a significant increase in ICU mortality and patients were grouped accordingly (ICU mortality: tBili ≤ 3.6 mg/dl: 46% (*n* = 273) vs. tBili > 3.6 mg/dl: 78% (*n* = 54), *p* < 0.001, Table 1). The Kaplan Meyer survival analysis for 28-day mortality depending on the initial Bili grouping revealed a significant difference notably within the first two weeks of treatment (hazard ratio (HR) 3.03 [2.07–4.43], *p* < 0.001, Fig. 2 A).

**Table 1:** Comparison of baseline characteristics between the groups

Characteristic	Initial tBili ≤ 3.6mg/dl (*n* = 273)	Initial tBili > 3.6mg/dl (*n* = 54)	*P* value
ICU mortality, n (%)	126 (46.2)	42 (77.8)	< 0.001
ECMO duration (days)	13.80 [6.30, 26.90]	6.75 [3.42, 14.12]	< 0.001
Age (years)	48.00 [38.00, 60.00]	48.50 [35.25, 62.75]	0.528
Male sex, n (%)	185 (67.8)	31 (57.4)	0.158
Body mass index (kg/m^2^)	24.69 [21.22, 29.39]	27.78 [24.49, 33.15]	0.003
PBW (kg)	70.46 [60.53, 74.99]	70.46 [56.91, 74.99]	0.630
Charlson comorbidity index	2.00 [1.00, 4.00]	3.00 [2.00, 5.00]	0.026
Chronic liver disease, n (%)	33 (12.1)	15 (27.8)	0.006
Immunocompromised, n (%)	68 (24.9)	18 (33.3)	0.236
SOFA at ARDS onset	11.00 [9.00, 14.00]	16.00 [13.00, 18.00]	< 0.001
APACHE at ARDS onset	28.00 [22.00, 34.00]	29.50 [20.00, 36.00]	0.712
SAPS at ARDS onset	55.00 [39.00, 69.00]	67.00 [56.25, 79.00]	< 0.001
RASS at ARDS onset	−5.00 [−5.00, −4.00]	−5.00 [−5.00, −4.12]	0.092
Pulmonary origin, n (%)	249 (91.2)	41 (75.9)	0.003
ARDS severity, n (%)			0.271
Mild	1 (0.4)	1 (1.9)	
Moderate	19 (7.0)	2 (3.7)	
Severe	253 (92.7)	51 (94.4)	
ARDS etiology, n (%)			0.002
Pneumonia	193 (70.7)	27 (50.0)	
Aspiration	20 (7.3)	12 (22.2)	
Other	60 (22.0)	15 (27.8)	
ECMO initiation (ICU day)	1.00 [1.00, 1.00]	1.00 [1.00, 1.00]	0.477
Mean ECMO blood flow (l/min)	3.55 [2.96, 4.15]	3.73 [3.26, 4.42]	0.133
Ventilation parameters at ECMO Initiation			
PaO_2_/FiO_2_ (mmHg)	68.90 [54.65, 91.10]	59.70 [52.50, 71.70]	0.009
PaO_2_ (mmHg)	67.60 [54.22, 89.52]	59.00 [52.50, 69.40]	0.016
PaCO_2_ (mmHg)	71.50 [56.08, 88.03]	60.90 [53.80, 68.40]	0.007
pH	7.23 [7.15, 7.31]	7.22 [7.18, 7.27]	0.755
PIP (cmH_2_O)	38.00 [34.00, 43.00]	41.64 [38.86, 47.00]	0.001
Pplat (cmH_2_O)	36.00 [31.25, 38.82]	36.69 [34.64, 40.50]	0.059
PEEP (cmH_2_O)	18.00 [14.00, 20.00]	21.00 [19.00, 23.10]	< 0.001
Driving pressure (cmH_2_O)	17.21 [13.00, 21.00]	17.30 [14.63, 21.50]	0.653
Respiratory rate (breaths/min)	25.00 [22.00, 28.00]	25.00 [21.00, 27.38]	0.903
Compliance (ml/cmH_2_O)	32.79 [21.00, 47.64]	30.33 [21.67, 40.87]	0.436
Tidal volume (ml)	377.73 [295.74, 491.43]	386.31 [255.04, 500.87]	0.883
Septic shock, n (%)	166 (60.8)	45 (83.3)	0.002
RRT, n (%)	181 (66.3)	38 (70.4)	0.636
PRBC units transfused (number)	18.00 [9.00, 32.00]	19.00 [13.00, 34.50]	0.322
Lactate (mg/dl)	18.00 [11.00, 43.00]	29.50 [16.25, 94.50]	0.002
Initial Hb (g/dl)	10.20 [9.00, 11.80]	10.00 [8.90, 10.78]	0.164
Initial CFH (mg/dl)	7.00 [4.00, 12.00]	8.00 [5.00, 14.50]	0.175
Initial Hp (g/l)	1.70 [0.78, 2.72]	0.66 [0.26, 1.47]	0.002
Hp depletion, n (%)	169 (61.9)	40 (74.1)	0.122
Further rescue therapies, n (%)			
Inhaled nitric oxide	191 (70.0)	46 (85.2)	0.029
Prone positioning	195 (71.4)	45 (83.3)	0.091

Data are expressed as mean (SD), median (25%, 75% quartiles) or frequencies (%), as appropriate

*CFH* Cell-free hemoglobin, *SOFA* Sequential Organ Failure Assessment, *APACHE* Acute Physiology And Chronic Health Evaluation, *SAPS* Simplified Acute Physiology Score, *RASS* Richmond Agitation-Sedation Scale, *PIP* Peak Inspiratory Pressure, *Pplat* plateau pressure, *PEEP* Positive End-Expiratory Pressure, *RRT* Renal Replacement Therapy, *PRBC* Packed Red Blood Cells

Baseline characteristics of the patients are shown in Table 1. After including all significantly different baseline characteristics in a multivariate regression model with backward variable elimination for ICU mortality, initial tBili grouping remained significantly associated with mortality (Table 2). Remaining variables in the final model were Charlson Comorbidity Index (CCI), chronic liver disease, serum lactate, septic shock and the ventilatory parameters PaO_2_, PaCO_2_ and PEEP at ECMO initiation (Table 2). No significant collinearity was observed in this model (variance inflation factors < 1.39). For the low tBili group, a significant increase of tBili levels compared to initial tBili was observed from day 7 to day 22, which was not noted in the high tBili group (Fig. 2 B). The tBili levels of the high tBili group remained significantly higher until day 12.

**Table 2:** Explanatory variables in simple and multiple logistic regression for ICU mortality

	**Simple logistic regression**		**Multivariate logistic regression**	
Variable	OR [95% CI]	*P* value	OR [95% CI]	*P* value
tBili grouping	4.08 [2.11–8.42]	< 0.001	3.09 [1.21–8.45]	0.021
CCI	1.14 [1.04–1.26]	0.004	1.17 [1.02–1.35]	0.028
Chronic liver disease	1.14 [0.62–2.12]	0.676	0.64 [0.26–1.56]	0.327
Lactate	1.01 [1.00–1.01]	0.009	1.01 [1.00–1.03]	0.019
Septic shock	1.35 [0.85–2.13]	0.196	0.76 [0.36–1.59]	0.472
PaO_2_ at ECMO initiation	0.99 [0.99–1.00]	0.393	0.99 [0.99–1.00]	0.541
PaCO_2_ at ECMO initiation	1.01 [1.00–1.02]	0.023	1.02 [1.00–1.03]	0.023
PEEP at ECMO initiation	0.98 [0.92–1.04]	0.484	0.94 [0.88–1.01]	0.090

Eliminated variables: BMI, inhaled nitric oxide, pulmonary origin, ECMO duration, ARDS etiology, SOFA, SAPS, PIP, Pplat and PaO_2_/FiO_2_ at ECMO initiation

The multivariate logistic regression model for ICU mortality was used with backward variable selection according to AIC. The Nagelkerke R^2^ of the final model was 0.374

*tBili* total Bilirubin, *OR* Odds Ratio, *CI* Confidence Interval, *CCI* Charlson Comorbidity Index

**Fig. 3:**
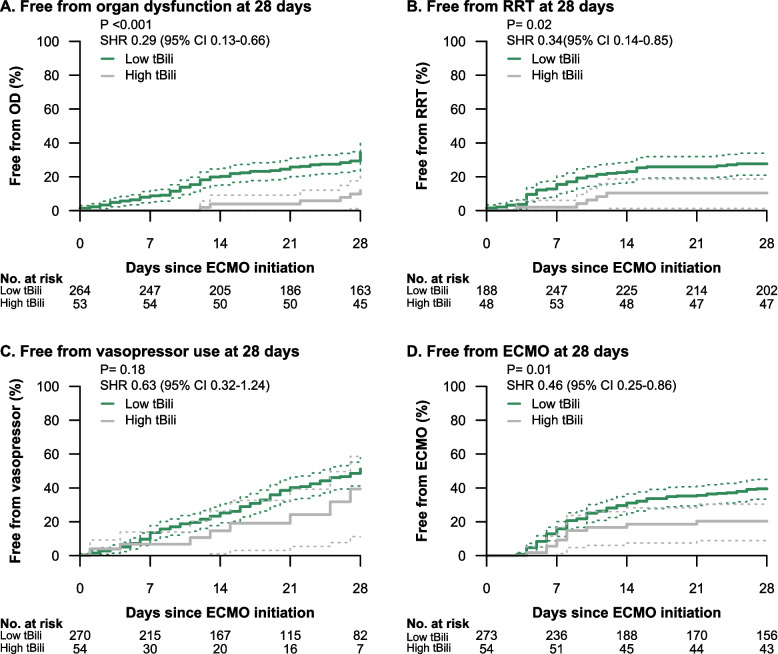
Cumulative incidence curves within 28 days for recovery from **A** organ dysfunction (subdistribution hazard ratio (SHR) 0.29 (95% CI 0.13–0.66), *P* < 0.001), **B** renal replacement therapy (RRT) (SHR 0.34 (95% CI 0.14–0.85), *P* = 0.02), **C** vasopressor use (SHR 0.63 (95% CI 0.32–1.24), *P* = 0.18) and **D** ECMO (SHR 0.46 (95% CI 0.25–0.86), *P* = 0.01)

For secondary endpoints recovery from organ dysfunction (SHR 0.29 [95%CI 0.13–0.66], *p* < 0.001), successful weaning from RRT (SHR 0.34 [0.14–0.85], *p* = 0.02), and successful weaning from ECMO (SHR 0.46 [0.25–0.86], *p* = 0.015), patients with an initial tBili ≤ 3.6 mg/dl plasma concentration showed significantly better outcomes compared to patients with an initial tBili > 3.6 mg/dl (Fig. 3 A-C). These differences remained significant after adjusting for the previously identified variables CCI, chronic liver disease, septic shock at ECMO initiation, and serum lactate (Supplemental Table 1). Patient groups did not differ in the secondary endpoint “successful weaning from vasopressor therapy” (SHR 0.63 [0.32–1.24], *p* = 0.18, Fig. 3 D).

## Discussion

In a cohort of 327 patients with ARDS and treatment with venous-venous ECMO, patients with an initial tBili plasma concentration over a threshold of 3.6mg/dl had significantly lower chances of survival, recovery from organ dysfunction, successful weaning from RRT, and successful weaning from ECMO within 28 days.

In a previous study, Zhai and colleagues observed a significantly higher mortality in patients with ARDS and a tBili plasma concentration above 4mg/dl at ICU admission [28]. Analogously, a correlation of initial tBili and mortality in ICU patients (initial tBili in survivors: 1.1mg/dl vs. initial tBili in deceased: 2mg/dl, *p* = 0.003) treated with veno-arterial ECMO for cardiac failure was observed by Freundt and colleagues [29]. While the study of Zhai and colleagues included patients with ARDS but without treatment with ECMO, Freudt and coworkers analyzed patients without ARDS but treated with veno-arterial ECMO [28, 29]. Pappalardo et al. designed the so-called ECMOnet Score for prediction of mortality in patients with ARDS due to H1N1 influenza A and treatment with veno-venous ECMO [30]. Besides Pre-ECMO hospital length of stay, serum creatinine, hematocrit, and mean arterial pressure, tBili was one of the relevant parameters of extrapulmonary organ function with strong correlation with survival [30]. Our findings are in line with these studies. However, in this study the prognostic value of tBili in a cohort of patients with ARDS and treatment with veno-venous ECMO was demonstrated. Analogous to the results of our study, Freundt and colleagues also observed a rise of tBili from day 2 of ECMO treatment onward, suggesting ongoing hemolysis during treatment with ECMO [2, 29]. Previous studies have demonstrated that treatment with veno-arterial ECMO systems induce even higher levels of hemolysis compared to veno-venous ECMO systems [2]. In a recent smaller retrospective study, an association between increased peak tBili plasma concentrations and 28-day mortality in patients with ARDS and treatment with ECMO was reported [17]. In this study of patients with ARDS and treatment exclusively with veno-venous ECMO, the increasing tBili values, most notably the increase in the low tBili group from day 7 to day 22, may reflect the combined impact of the underlying disease and the treatment with an extracorporeal organ replacement system, promising outcome predictor in patients with ARDS and treatment with veno-venous ECMO.

While the initial plasma concentration of CFH did not differ between the groups, significant differences in initial Hp plasma levels were detected. Specifically, patients with increased initial tBili plasma concentrations had decreased Hp plasma levels. However, incidence of haptoglobin depletion did not differ between the groups. This finding may reflect a degree of hemolysis that has not led to an overload of the Hp scavenger system at the time of ECMO initiation, allowing control of the intravascular concentrations of CFH [24]. Although patients with an initial tBili above 3.6mg/dl had a significantly higher rate of chronic liver disease compared to patients with an initial tBili below 3.6mg/dl, the multivariate regression analysis did not reveal a significant association between mortality and chronic liver disease.

The study is limited by the retrospective and single center design. However, to our knowledge, this analysis comprises one of the largest cohorts of patients with severe ARDS and exclusive treatment with veno-venous ECMO. This allows an acceptable statistical power and a higher homogeneity compared to previous studies investigating the prognostic potential of tBili plasma concentrations in patients with ARDS and treatment with ECMO [16, 17]. Whether the increased tBili plasma concentrations are generated from reduced tBili metabolism, reduced tBili excretion, or from increased CFH metabolism due to ongoing hemolysis in patients with ARDS and treatment with veno-venous ECMO remains unclear from these retrospective data. Future research should explore the biochemical mechanisms how tBili and breakdown products of hemolysis impact organ function. In addition, the relevance of increased CFH metabolism for increased production of tBili should be investigated. In addition, a prospective study to confirm the prognostic role of initial tBili levels in predicting ICU mortality and recovery from organ dysfunction would be necessary to confirm the results obtained in the current study. With potential availability of a supplementation therapy with exogenous haptoglobin, CFH-associated adverse effects might be ameliorated [31]. Furthermore, it is unclear whether the pathophysiological direct toxic effect of elevated tBili plasma concentrations may have directly or just indirectly contributed to the observed associations with mortality and organ dysfunction [13, 23, 28, 32, 33]. Rather, tBili might just serve as a surrogate parameter for liver dysfunction and hemolysis in patients with ARDS and treatment with veno-venous ECMO. Adjustments for secondary liver dysfunctions induced by hypoxia, toxins, or drugs and for the use of different ECMO protocols, hemadsorption therapy, other potentially hemolysis-affecting medication or immunologic factors were not made in this study [34]. In addition, no specific hemodynamic data were included in the analyses of this study and no adjustments could be made for e.g. right heart failure which can lead to hepatic congestion and consecutive compromised liver function.

## Conclusions

Patients with ARDS, treatment with veno-venous ECMO and a tBili plasma concentration over the threshold of 3.6 mg/dl had significantly lower chances of survival, recovery from organ dysfunction, successful weaning from RRT, and successful weaning from treatment with ECMO within 28 days compared to patients with a tBili plasma concentration below 3.6 mg/dl. The identified tBili-cut-off value might help to identify patients with ARDS and treatment with veno-venous ECMO with a high risk of unfavorable outcomes.

## Supplementary Information

Supplementary Material 1.

## Data Availability

Data are available from the corresponding author on reasonable request.

## Abbreviations

ARDS: Acute respiratory distress syndrome
CFH: Cell-free hemoglobin
Hp: Haptoglobin
tBili: Total bilirubin
NO: Nitric oxide
V-V ECMO: Veno-venous extracorporeal membrane oxygenation
CCI: Charlson comorbidity index
BMI: Body mass index
SOFA: Sequential organ failure assessment
APACHE: Acute physiology and chronic health evaluation
SAPS: Simplified acute physiology score
RASS: Richmond agitation-sedation scale
PIP: Peak inspiratory pressure
Pplat: Plateau pressure
PEEP: Positive end-expiratory pressure
RRT: Renal replacement therapy
PRBC: Packed red blood cells
SHR: Subdistribution hazard ratio
