# Supplementary material for: Total bilirubin as a marker for hemolysis and outcome in patients with severe ARDS treated with veno-venous ECMO
Source: BMC Anesthesiol. 2025 Mar 13;25:121. doi: 10.1186/s12871-025-02988-1 (PMC11905513; doi:10.1186/s12871-025-02988-1)
Supplement: Supplementary file 1 — Supplementary Material 1. [file 12871_2025_2988_MOESM1_ESM.docx]

# Supplementary Material

# Total bilirubin as a marker for hemolysis and outcome in patients with severe ARDS treated with veno-venous ECMO

Victoria Bünger, Mario Menk, Oliver Hunsicker, Alexander Krannich, Felix Balzer, Claudia D. Spies, Wolfgang M. Kuebler, Steffen Weber-Carstens, Jan A. Graw

# Supplemental Methods

## Data Sources

Data on patient demographics, ARDS etiology and comorbidities were extracted from the hospital data management system (SAP, Walldorf, Germany). Further data regarding admission scores, ARDS characteristics, treatment, rescue therapies and medication, ventilation parameters and laboratory parameters were extracted from the electronic ICU data management system used at the hospital (COPRA 5, Sasbachwalden, Germany). Daily measurements of CFH, Hp plasma and total bilirubin concentrations in patients with ARDS and therapy with veno-venous ECMO were obtained from EDTA blood for standard laboratory tests. Blood was centrifuged (15 min, 2500 g) and analyzed by a COBAS 8000 modular analyzer series system (Roche Diagnostics, Basel) as described previously (1).

1. Graw JA, Hildebrandt P, Krannich A, et al. The role of cell-free hemoglobin and haptoglobin in acute kidney injury in critically ill adults with ARDS and therapy with VV ECMO. *Crit Care*. 2022;26(1):50. doi:10.1186/s13054-022-03894-5

# Supplemental Tables

### Supplemental Table 1: Subdistribution Hazard Ratios adjusted for CCI, septic shock, chronic liver disease and lactate.

| Secondary Endpoint | Adj. SHR [95% CI] | P value |
| --- | --- | --- |
| Free from OD | 0.37 [0.16-0.84] | 0.019 |
| Free from RRT | 0.38 [0.15-0.98] | 0.045 |
| Free from vasopressor use | 0.68 [0.34-1.35] | 0.276 |
| Free from ECMO | 0.48 [0.25-0.90] | 0.023 |
| Definition of abbreviations: OD – organ dysfunction. RRT – renal replacement therapy. SHR – subdistribution hazard ratio. CI – confidence interval. | | |
